# Drug repurposing for disease-modifying effects in multiple system atrophy

**DOI:** 10.1186/s40035-026-00551-7

**Published:** 2026-04-21

**Authors:** Seong Ho Jeong, Jin Young Shin, Phil Hyu Lee

**Affiliations:** 1 Department of Neurology, Yonsei University College of Medicine, 50 Yonsei-ro, Seodaemun-gu, Seoul, 03722 South Korea; 2 Department of Neurology, Yongin Severance Hospital, Yonsei University Health System, Yongin, South Korea; 3 Severance Biomedical Science Institute, Yonsei University College of Medicine, Seoul, South Korea

**Keywords:** Multiple system atrophy, Drug repurposing, α-Synuclein, Neurodegeneration

## Abstract

Multiple system atrophy (MSA) is a rapidly progressive neurodegenerative disorder lacking any proven disease-modifying therapy. Drug repurposing offers a strategy to accelerate the development of treatments by utilizing agents originally approved for other indications. This review summarizes repurposed drugs investigated as disease-modifying therapies for MSA, spanning preclinical in vitro and animal studies and clinical trials. We focus on agents targeting key pathogenic mechanisms in MSA—including α-synuclein aggregation (e.g., sirolimus/rapamycin, rifampicin, lithium, nilotinib, epigallocatechin gallate), neuroinflammation (e.g., minocycline, intravenous immunoglobulin), mitochondrial dysfunction and excitotoxicity (e.g., ubiquinol, rasagiline, safinamide, riluzole), and impaired neurotrophic support (e.g., fluoxetine/selective serotonin reuptake inhibitors, insulin, exendin-4). For each, we discuss mechanisms of action, experimental model outcomes, and clinical trial results. While numerous repurposed agents showed promise in MSA models, most failed to demonstrate significant disease-slowing effects in clinical trials. However, ubiquinol has recently emerged as a notable exception, with a Phase 2 randomized controlled trial showing a significant reduction in motor progression compared to placebo—marking the first placebo-controlled evidence of disease modification in MSA. Limitations such as small sample sizes, late-stage patient enrollment, and tolerability issues (e.g., with lithium) have hampered past trials. Nonetheless, ongoing studies and emerging approaches such as combination therapies hold promise. Continued exploration of repurposed therapies, along with improved trial design and biomarker development, is warranted to finally achieve a disease-modifying treatment for MSA.

## Introduction

Multiple system atrophy (MSA) is an adult-onset, fatal neurodegenerative disease characterized by a variable combination of parkinsonism, cerebellar ataxia, and autonomic failure [1, 2]. Pathologically, MSA is defined by the accumulation of misfolded α-synuclein proteins within oligodendroglial cells, forming glial cytoplasmic inclusions (GCIs). These toxic aggregates are thought to trigger a cascade of oligodendroglial dysfunction, myelin disruption, and widespread neurodegeneration [3]. The α-synuclein pathology is accompanied by prominent neuroinflammation, including microglial activation and astrogliosis with release of pro-inflammatory cytokines and oxidative species [3, 4]. The result is progressive neuronal loss in striatonigral and olivopontocerebellar circuits, leading to rapid clinical deterioration. MSA patients typically reach severe disability within 5–10 years of symptom onset, and effective disease-modifying therapies are a critical unmet need [5–7]. Unlike Parkinson’s disease (PD), which shares the α-synuclein pathology, MSA shows poor or transient response to dopaminergic therapies, and no medication is known to slow its relentless course.

Over the past two decades, numerous agents with neuroprotective potential have been tested in MSA based on insights from related neurodegenerative diseases (e.g. PD, Alzheimer’s disease, and amyotrophic lateral sclerosis) and from preclinical models. These efforts have targeted major pathogenic pathways in MSA, including: (1) aggregation and clearance of α-synuclein, (2) neuroinflammation and glial dysfunction, (3) mitochondrial impairment, oxidative stress, and excitotoxicity, and (4) loss of neurotrophic support. Unfortunately, despite encouraging results in laboratory models, none of the candidate disease-modifying therapies tested in large trials thus far have proven effective in slowing MSA progression. This translational gap underscores challenges such as the aggressive course of MSA (difficulty in early intervention), limited patient numbers for trials, and model systems that only partially recapitulate human disease.

**Fig. 1:**
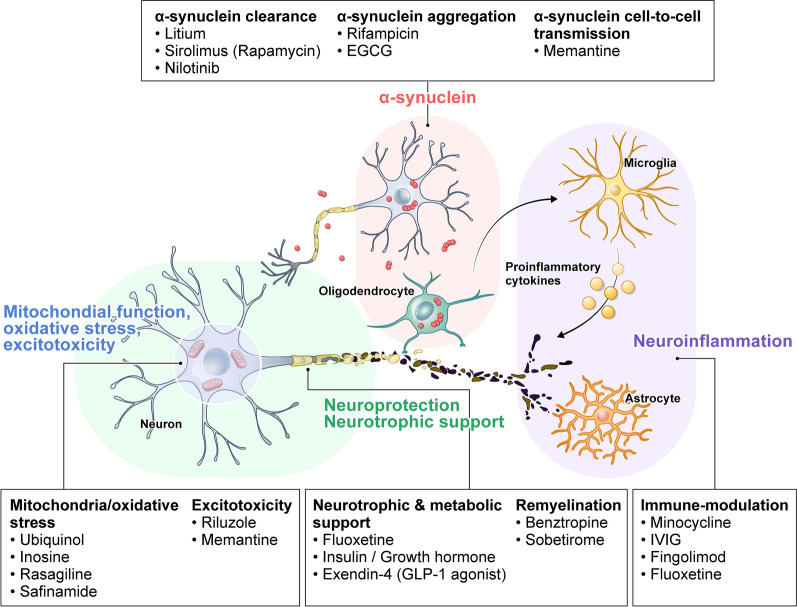
Key pathological mechanisms and therapeutic targets in multiple system atrophy. Misfolded α-synuclein (red) accumulates in oligodendrocytes, forming glial cytoplasmic inclusions and contributing to neuronal dysfunction. This aggregation triggers secondary neuroinflammation from activated microglia and astrocytes (purple area), which release proinflammatory cytokines that exacerbate neurodegeneration. Meanwhile, affected neurons (green area) suffer loss of trophic support and other toxic stresses. Mitochondrial dysfunction, oxidative stress, and excitotoxicity act in concert to disrupt neuronal homeostasis, leading to energy failure, accumulation of reactive oxygen species, and glutamate-mediated toxicity that collectively accelerate neurodegeneration. Experimental therapies targeting each of these pathological mechanisms—identified through drug repurposing strategies—are also depicted in the figure, illustrating how specific agents aim to modulate α-synuclein aggregation, neuroinflammation, mitochondrial dysfunction, oxidative stress, excitotoxicity, and neurotrophic deficiency

Drug repurposing (or repositioning) has emerged as an attractive strategy to identify potential MSA treatments. By using drugs already approved for other indications, this approach can bypass early-stage safety testing and accelerate the path to clinical trials [8]. Many repurposed agents also have well-characterized mechanisms that align with the MSA pathology, providing a strong rationale for their use. This review provides a structured overview of drug repurposing efforts for disease modification in MSA. We focus on agents originally developed for other diseases—including several already approved drugs—that have shown potential to modify MSA pathology or progression in preclinical studies or clinical trials. We discuss each candidate’s mechanism of action, evidence from in vitro and animal models, outcomes of clinical studies in MSA, and any limitations observed. Finally, we consider the lessons learned and future directions for advancing disease-modifying therapy in MSA. Figure 1 categorizes the following therapies based on their primary target mechanisms. This classification is intended to provide an organizational framework for major pathogenic pathways in MSA; however, many agents exert pleiotropic effects and may influence multiple mechanisms, resulting in overlap between categories.

## Repurposed therapeutic strategies in MSA

### Enhancing α-synuclein clearance and proteostasis

Abnormal accumulation of α-synuclein is a central feature of MSA, making its clearance a prime therapeutic target [9]. Several repurposed compounds that modulate protein degradation pathways—particularly autophagy and ubiquitin–proteasome systems—have been investigated to enhance α-synuclein removal. Drugs targeting this mechanism, along with their mechanisms of action, preclinical evidence, clinical trial outcomes, and current status, are summarized in Table 1.

**Table 1:** Summary of therapies targeting enhanced α-synuclein clearance and proteostasis in multiple system atrophy

Drug/Therapy	Original indication	Mechanism of action	Preclinical evidence	Clinical trial outcome	Current status
Sirolimus (rapamycin)	Immunosuppressant (organ transplant rejection)	mTOR inhibitor; induces autophagy, promotes clearance of protein aggregates	Yes – MSA transgenic mice: autophagy ↑, α-synuclein inclusions ↓, partial neuroprotection	Phase II futility trial in MSA: no slowing of progression vs. placebo (terminated early for futility)	Trial halted; no further development for MSA (not effective)
Rifampicin	Antibiotic (tuberculosis, leprosy)	Binds α-synuclein; inhibits fibril formation and disaggregates existing fibrils; also anti-inflammatory properties	Yes – MSA mice: long-term treatment decreased α-syn levels and neurodegeneration	Phase III trial in early MSA: no difference in motor decline vs. placebo; stopped early for futility	Development discontinued for MSA (no efficacy in trial)
Lithium	Mood stabilizer (bipolar disorder)	Inhibits GSK-3β and inositol monophosphatase; promotes autophagy and neurotrophic signaling; anti-apoptotic effects	Yes – Parkinsonian models: autophagy ↑, neuroprotective effects (reduced aggregates)	Phase II trial in MSA terminated early due to toxicity (several serious adverse effects); no evidence of benefit (trial halted)	Not pursued further due to safety concerns (narrow therapeutic window in MSA)
Nilotinib	Tyrosine kinase inhibitor (chronic myelogenous leukemia)	Inhibits c-Abl tyrosine kinase; activates lysosomal clearance/autophagy of misfolded proteins; reduces oxidative stress	Yes – PD models: α-syn clearance ↑, oxidative stress ↓; MSA mice: no significant motor or pathology improvement	No completed MSA trials to date; small open-label in PD/LBD suggested reduced CSF α-syn and tau; Phase II PD showed no clinical benefit and poor CNS penetration at safe dose	Investigational – interest remains but efficacy unproven; challenges with brain delivery and toxicity (QT prolongation)
Memantine	NMDA receptor antagonist (Alzheimer’s disease dementia)	Blocks NMDA glutamate receptors (reduces excitotoxicity); may inhibit cell-to-cell spread of α-syn (reduces internalization and propagation of α-synuclein)	Yes – PD models: α-syn propagation and neuronal loss ↓, improved motor function; concept applicable to MSA (α-syn spread is a key pathological feature)	No clinical trials in MSA yet (safety well-established from AD use; potential benefit speculative)	Investigational – proposed for MSA but not yet tested; considered for early-stage intervention to prevent pathology spread
Epigallocatechin-3-gallate (EGCG)	Green tea polyphenol supplement	Binds misfolded proteins; prevents toxic oligomer/fibril formation; redirects proteins to non-toxic aggregates; antioxidant	Yes – in vitro: prevents α-syn toxic aggregation; antioxidant effects (preclinical rationale for trial)	Phase II/III trial (PROMESA) in MSA (1200 mg/day): no significant slowing of progression vs. placebo; some hepatotoxicity observed at high dose	Not effective at high dose; use in MSA not supported (potential liver toxicity risk)

MSA, multiple system atrophy; PD, Parkinson’s disease; LBD, Lewy body dementia; GCIs, glial cytoplasmic inclusions; mTOR, mechanistic target of rapamycin; GSK-3β, glycogen synthase kinase-3 beta; CNS, central nervous system; NMDA, N-methyl-D-aspartate

#### Sirolimus (rapamycin)

Sirolimus (also known as rapamycin) is an immunosuppressant originally used to prevent organ transplant rejection, now repurposed for its autophagy-inducing properties. Mechanistically, sirolimus allosterically inhibits the mammalian target of rapamycin (mTOR) kinase, a key negative regulator of autophagy [10]. By blocking mTOR, sirolimus promotes autophagic degradation of proteins and organelles [11]. In preclinical models of synucleinopathy, including a transgenic mouse model overexpressing α-synuclein in oligodendrocytes (a model of MSA), sirolimus treatment increased the autophagic flux, reduced α-synuclein aggregates, and provided partial neuroprotection [11, 12]. MSA mice treated with sirolimus showed lower glial inclusion burden and some preservation of neurons, supporting the hypothesis that enhancing lysosomal clearance can mitigate pathology [12, 13]. Encouraged by these findings, a single-site phase II randomized, double-blind, placebo-controlled futility trial of oral sirolimus (2–6 mg/day) was conducted in probable MSA to test disease-slowing over 48 weeks [14]. A total of 47 patients were randomized (35 sirolimus, 12 placebo), but the study was halted after a pre-planned interim futility analysis. The primary endpoint—change in the Unified MSA Rating Scale (UMSARS) total from baseline to week 48—showed no benefit of sirolimus versus placebo (13.92 vs. 11.30; difference 2.66; 95% CI − 7.35 to 6.91; *P* = 0.648). UMSARS-I (activities of daily living) and UMSARS-II (motor) were likewise similar (UMSARS-I: 5.65 vs. 5.33; *P* = 0.707; UMSARS-II: 6.58 vs. 6.50; *P* = 0.996). Secondary outcomes—including brain magnetic resonance imaging (MRI) (putaminal mean diffusivity/volumes and other volumetrics), retinal optical coherence tomography, and plasma biomarkers (neurofilament light chain [NfL]; neuronal/oligodendroglial α-syn exosomes)—showed no between-group differences, while adverse events (AEs) such as infections, oral lesions, diarrhea, edema, and skin eruptions were more frequent with sirolimus; in pooled longitudinal analyses, plasma NfL increases and whole-brain atrophy correlated with UMSARS worsening. Sirolimus was reasonably well tolerated immunologically, but isolated reports suggested that chronic mTOR inhibition might paradoxically activate certain immune responses (e.g., increased IL-6 release from microglia) [10], highlighting the complexity of its effects. Overall, while sirolimus successfully induced autophagy in animal models, this did not translate into measurable clinical improvement in the treated MSA cohort. Other possible reasons may include issues of disease stage (very early stage or prodromal stage of MSA) or the presence of autophagy-independent disease mechanisms.

#### Rifampicin

Rifampicin is a broad-spectrum antibiotic (widely used for tuberculosis) that was repurposed for synucleinopathies after the discovery of its anti-aggregation effects on α-synuclein. In vitro, rifampicin can bind α-synuclein and inhibit its fibrillation, as well as promoting disaggregation of preformed fibrils [15]. In MSA model mice, long-term rifampicin treatment was reported to reduce brain α-synuclein levels and attenuate neurodegeneration [16]. The proposed mechanisms include enhancement of intracellular degradation pathways and direct interference with toxic oligomer formation​. These promising preclinical actions led to a multicenter Phase III randomized, double-blind, placebo-controlled trial of oral rifampicin (300 mg twice daily) in patients with early MSA [17]. A total of 100 patients were randomized (50 rifampicin, 50 placebo) and followed for 12 months. The primary outcome was the rate of change in UMSARS-I. An interim futility analysis (15 patients/group) showed no benefit, leading to early termination. Final analysis of 49 rifampicin-treated and 50 placebo-treated patients confirmed no disease-modifying effect: the slope of UMSARS-I was identical (0.5 points/month in both groups; *P* = 0.82), and absolute changes in UMSARS-I (6.2 in the treatment group vs. 5.6 in the placebo group; *P* = 0.62), UMSARS II (7.0 in the treatment group vs. 5.4 in the placebo group; *P* = 0.23), and total UMSARS (12.9 in the treatment group vs. 10.8 in the placebo group; *P* = 0.31) were also nonsignificant. Secondary outcomes, including autonomic symptoms (Composite Autonomic Symptom Score (COMPASS)-select and COMPASS-select-change), also showed no improvement with rifampicin. Although AEs were frequent, serious AEs occurred in fewer rifampicin patients (6%) than in placebo-treated patients (24%), and none were considered treatment-related. This negative result tempered the initial enthusiasm from bench studies. Notably, the antibiotic activity of rifampicin necessitated relatively a short trial duration (to avoid resistance and side effects), which may have been insufficient if a longer period is needed to affect neurodegeneration. Furthermore, achieving adequate brain levels of rifampicin in humans is challenging, given that cerebrospinal fluid (CSF) concentrations represent only 5% of plasma levels [18, 19].

#### Lithium

Lithium carbonate, long used as a mood stabilizer in bipolar disorder, has multiple neurobiological effects that made it a candidate for repurposing in synucleinopathies. Of relevance, lithium can induce autophagy by inhibiting glycogen synthase kinase-3 beta (GSK-3β) and inositol monophosphate, thereby promoting clearance of protein aggregates [20, 21]. Lithium also has anti-apoptotic and neurotrophic signaling effects. In cellular and rodent models of parkinsonian disorders, lithium reduced α-synuclein aggregation and protected neurons, partly via autophagy upregulation [22, 23]. These multimodal actions prompted a single-center, phase II, randomized, double-blind, placebo-controlled pilot trial of oral lithium carbonate in MSA, planned for 48 weeks. Lithium was initiated at 150 mg twice daily and titrated in 300-mg increments up to 1500 mg/day to target serum 0.9–1.2 mmol/L [24]. Nine patients were randomized (four lithium, five placebo; 10 enrolled). The primary endpoint was safety/tolerability, with secondary outcomes including disease progression assessed by UMSARS, brain magnetic resonance spectroscopy, EuroQol 5-Dimension Questionnaire (EQ-5D), and Beck Depression Inventory (BDI)-II depression scores. At an independent interim analysis (one year after first randomization), the trial was terminated early for safety: premature treatment discontinuation or death occurred in 100% of lithium-treated patients (4/4) vs. 20% of placebo-treated patients (1/5; *P* < 0.01), and the adverse-event burden was higher with lithium after adjusting for exposure (58.4 in the treatment group vs. 21 in the placebo group; *P* < 0.02). Since no lithium-treated patient reached the 24-week reassessment, planned UMSARS change analyses (Part I, Part II, or total) were not assessable, and no between-group UMSARS change can be reported. Lithium’s narrow therapeutic window and vulnerability to dehydration or drug interactions posed particular hazards in an autonomic disorder like MSA. Thus, despite intriguing mechanistic rationale (autophagy enhancement and neuroprotection), the repurposing of lithium for MSA was halted due to tolerability issues.

#### Epigallocatechin-3-gallate (EGCG)

Besides the above, several other agents originally developed for different conditions have been tested for anti-aggregation effects in MSA. EGCG, a green tea polyphenol marketed as a supplement, is one prominent example. EGCG can bind misfolded proteins and prevent their formation into toxic oligomers or fibrils [25, 26]. In vitro, EGCG redirects α-synuclein into off-pathway aggregates, which are defined as alternative assemblies that deviate from the canonical fibrillization pathway and are non-toxic, and exhibits antioxidant properties [27, 28]. On this basis, a multicenter Phase II/III PROMESA trial tested oral EGCG in MSA, titrated to 1200 mg/day (400 mg once daily for 4 weeks → twice daily for 4 weeks → three times daily for 40 weeks), with 48 weeks of treatment plus a 4-week washout (total 52 weeks) [29]. A total of 92 patients with MSA were randomized (47 EGCG, 45 placebo). The primary endpoint—change from baseline to week 52 in UMSARS Part II (motor exam)—showed no significant benefit (5.66 in the treatment group vs. 6.60 in the placebo group; difference − 0.94, 95% CI − 3.71 to 1.83; *P* = 0.51). The total UMSARS change likewise did not differ (10.33 in the treatment group vs. 10.35 in the placebo group; *P* = 0.99). Secondary outcomes (Clinical Global Impression [CGI]) were also negative, while an exploratory MRI sub-study suggested less atrophy of striatal and precentral gyrus with EGCG and, among participants in the MRI sub-study who completed the trial according to protocol, a larger reduction in UMSARS Part II (mean difference − 5.18; *P* = 0.014). Hepatotoxicity was more frequent with EGCG (elevated aminotransferases in 17% overall; two discontinuations for liver toxicity), and serious AEs occurred more often in EGCG than in placebo (29 vs. 14), with deaths of 4 vs. 2 during the study [30]. Thus, despite being generally safe as a dietary supplement, pharmacologic doses of EGCG are not beneficial and may carry risks, underscoring the importance of rigorous trials even for “natural” compounds.

#### Future candidates under this mechanism

Nilotinib is a tyrosine kinase inhibitor originally developed for chronic myelogenous leukemia (targeting BCR-Abl). It received attention in neurodegeneration when preclinical studies showed nilotinib could boost clearance of misfolded proteins by activating the lysosomal-autophagy pathway [31, 32]. In models of PD, nilotinib increases the breakdown of α-synuclein and reduces oxidative stress markers [32, 33]. These effects are thought to arise from inhibition of Abl kinase activity [34], which in turn enhances autophagic flux and may stabilize microglial activation. Nilotinib thus represents a high-potency small molecule approach to inducing intracellular protein clearance, distinct from mTOR inhibition. However, an initial study in transgenic MSA mice showed a lack of preclinical efficacy, as nilotinib did not significantly ameliorate motor deficits or α-synuclein-related pathology in these models [35]. To date, however, no human clinical studies of nilotinib have been conducted in patients with MSA. Nonetheless, in other α-synucleinopathies, a small open-label trial in patients with advanced PD and Lewy body disease suggested that nilotinib (at low doses) is reasonably safe and reduces central neurodegenerative biomarkers (with decreased CSF α-synuclein oligomers and tau) [36]. A subsequent phase II PD trial, however, found that nilotinib did not produce clinical improvement and achieved only minimal CSF penetration at tolerated doses [37]. This raised doubts about its viability for brain proteinopathies. The mixed outcomes imply that while nilotinib can engage autophagy pathways, it is challenging to achieve sufficient brain levels without systemic toxicity. Future modifications of nilotinib or use of alternative Abl inhibitors with better brain permeability may be needed to achieve improved efficacy in synucleinopathies.

Memantine, an N-methyl-D-aspartate (NMDA) receptor antagonist approved for Alzheimer’s disease, has shown potential in modulating α-synuclein transmission, relevant to MSA pathology. In PD models, our previous study demonstrated that memantine decreases internalized cytosolic α-synuclein and attenuates α-synuclein-induced cell death [38]. It inhibited extracellular α-synuclein propagation, reducing phosphorylated α-synuclein in dopaminergic neurons and improving motor function, suggesting a role in preventing pathological spread. In MSA, α-synuclein spreading is critical, particularly among oligodendrocytes, where it forms GCIs. The spreading of α-synuclein is well-known in PD. While GCI-specific transmission in MSA remains less understood, similar mechanisms likely contribute to disease progression. A recent study showed that oligodendrocytes, but not neurons, can generate distinct α-synuclein strains, suggesting that environmental differences may drive pathology [39]. Given this, the ability of memantine to block α-synuclein transmission could be beneficial, though direct MSA studies are lacking. The safety profile of memantine has been well-established, with common side effects like dizziness and headache, making it a candidate for repurposing. However, its efficacy in MSA requires further investigation, potentially through trials focusing on early-stage patients to target spreading before extensive neuronal loss.

### Modulating neuroinflammation

Neuroinflammation is heavily implicated in MSA pathogenesis, as activated microglia and astrocytes are found in affected brain regions and may worsen neuronal death [40]. Drugs with anti-inflammatory or immunomodulatory actions have therefore been repurposed in attempts to slow MSA. These agents aim to dampen harmful glial activation or autoimmune processes that could contribute to MSA progression. Table 2 outlines drugs associated with neuroinflammation, describing their mode of action, preclinical data, clinical trial findings, and ongoing development status.

**Table 2:** Summary of therapies targeting neuroinflammation in multiple system atrophy

Drug/Therapy	Original indication	Mechanism of action	Preclinical evidence	Clinical trial outcome	Current status
Minocycline	Antibiotic (tetracycline class)	Inhibits microglial activation and proliferation; anti-inflammatory and anti-apoptotic effects; crosses BBB	Yes – various neurodegenerative models: neuroprotection; MSA-P rat model: suppressed microglial activation but no prevention of neuron loss	Phase II trial (MEMSA, 1 year): no improvement in UMSARS vs. placebo; PET showed reduced microglial activation (target engagement); well tolerated	Safe but no clinical efficacy; not actively pursued further as monotherapy
Intravenous Immunoglobulin (IVIg)	Immunotherapy for autoimmune/inflammatory diseases	Pooled antibodies from donors; modulates immune response (neutralizes autoantibodies, inhibits complement, regulates T-cells and cytokines)	Limited – no specific MSA animal model; rationale from immune dysregulation observed in MSA	Open-label pilot (9 patients): suggested slower decline and some motor/autonomic improvements; well tolerated (no serious adverse events) – no placebo control	Investigational – preliminary positive signal but no RCT; no ongoing large trials (high cost & logistical challenges)
Fingolimod (FTY720)	Oral immunomodulator (multiple sclerosis)	S1P receptor modulator; sequesters lymphocytes (reduces peripheral inflammation); may boost CNS neurotrophic factors (BDNF, GDNF)	Yes – modified fingolimod (FTY720-mitoxy) in MSA mice: increases BDNF/GDNF/NGF, neuroprotection (improved motor, decreases neuroinflammation)	No trials in MSA patients yet (evidence limited to preclinical studies)	Investigational – potential repurposing candidate; not yet tested clinically in MSA

MSA, multiple system atrophy; PET, positron emission tomography; UMSARS, Unified MSA Rating Scale; BBB, blood-brain barrier; S1P, sphingosine-1-phosphate; BDNF, brain-derived neurotrophic factor; GDNF, glial cell line-derived neurotrophic factor; NGF, nerve growth factor

#### Minocycline

Minocycline, a second-generation tetracycline antibiotic, has notable anti-inflammatory and neuroprotective properties distinct from its antimicrobial use. It readily crosses the blood–brain barrier and inhibits microglial activation and proliferation [41, 42]. Minocycline has conferred neuroprotection and improved outcomes in models of various neurodegenerative diseases [41, 43, 44], which made it an appealing repurposed candidate for MSA. However, in a toxin-induced rat model of striatonigral degeneration (mimicking MSA-P), minocycline failed to prevent neuron loss despite suppression of microglial activation [45]. This hinted that inflammation might be a byproduct rather than a driver of neurodegeneration in that model. Nonetheless, a multicenter, randomized, double-blind, placebo-controlled Phase II trial (MEMSA) evaluated oral minocycline 100 mg twice daily (200 mg/day) over 48 weeks in 63 patients with MSA-P (32 minocycline, 31 placebo), and included advanced imaging to assess microglial activity (^11^C-PK11195 PET) [46]. The primary endpoint—change in UMSARS Part II from baseline to week 48—showed no benefit of minocycline: mean change 8.2 ± 6.3 vs. 7.0 ± 7.1 (minocycline vs. placebo; *P* = 0.175). Secondary outcomes (UMSARS I/III, UPDRS II–III, EQ-5D, 12-item Short Form Health Survey) likewise showed no between-group differences. In an exploratory ^11^C-PK11195 PET substudy (*n* = 8; 3 minocycline, 5 placebo), minocycline-treated patients showed attenuated increases in microglial activation (two of three showed decreased binding; Wilcoxon *P* = 0.07). Discontinuations were frequent (72% in the treatment group vs. 32% in the placebo group), and deaths were 3 vs. 2 during the study. In other words, minocycline hit its intended immunomodulatory target but this did not translate into functional gain within the trial period. Minocycline was generally well tolerated in MSA (with no major AEs reported in the trial), affirming its safety profile.

#### Intravenous immunoglobulin (IVIg)

IVIg – a pooled antibody preparation from healthy donors – is an established therapy in various autoimmune and inflammatory conditions. It exerts immunomodulatory effects by multiple mechanisms: IVIg can neutralize autoantibodies, inhibit complement activation, suppress pathogenic T-cell activity, and skew cytokine production toward an anti-inflammatory profile​ [47]. The rationale for IVIg use in MSA stemmed from reports of peripheral immune dysregulation in MSA and the general concept that dampening inflammation might protect neurons. A single-centre, open-label Phase II pilot study tested IVIg (0.4 g/kg monthly for 6 months) in nine patients with probable MSA (seven completers) [48]. The primary endpoint was safety/tolerability; secondary endpoints included changes in UMSARS and quantitative brain MRI. From baseline to post-treatment, UMSARS-I improved from 23.9 ± 6.0 to 19.0 ± 5.9 (difference − 4.9 points; *P* = 0.01) and UMSARS-II from 26.1 ± 7.5 to 23.3 ± 7.3 (difference − 2.8; *P* = 0.025), while UMSARS-III/IV showed no significant change. No serious AEs occurred; the most frequent infusion-related effect was transient blood-pressure elevation (e.g., systolic rise during infusions; *P* = 0.05), and two withdrawals due to pruritic rash. On imaging, quantitative MRI volumetry showed no significant pre- vs. post-treatment differences over an approximately 8-month interval (baseline and within ~ 1 month after the last infusion). Based on these encouraging findings, the authors called for a larger controlled trial. To date, a confirmatory randomized trial of IVIg in MSA has not been completed, likely due to logistical challenges and the cost. Thus, IVIg remains a tentative repurposed option – biologically plausible and with anecdotal benefit, but lacking high-level evidence. Future studies could include biomarkers of immune activation to pinpoint which patients might respond to the IVIg therapy.

#### Future candidates under this mechanism

Beyond minocycline and IVIg, researchers have considered other repurposed immunomodulatory therapies for MSA, though data are limited. One repurposed agent tested preclinically is lenalidomide, a derivative of thalidomide used in multiple myeloma that has potent anti-cytokine and microglia-suppressing properties. In a transgenic MSA mouse model, lenalidomide combined with an experimental α-synuclein antibody reduced neuroinflammation and α-synuclein levels more than either alone [49]. This suggests that immunomodulators like lenalidomide might boost the efficacy of immunotherapy, though side effects (e.g. bone marrow suppression) of lenalidomide could limit its clinical use in MSA.

Fingolimod, an oral sphingosine-1-phosphate receptor modulator approved for multiple sclerosis, is another immune-targeted therapy considered for repurposing. Fingolimod sequesters lymphocytes and also may directly promote neurotrophic factors in the brain [50]. A modified version of fingolimod (FTY720-mitoxy) increased brain-derived neurotrophic factor (BDNF), glial cell line-derived neurotrophic factor (GDNF), and NGF (nerve growth factor) expression and exerted neuroprotective effects in a mouse model of MSA​ [51]. This led to improved motor function and reduced neuroinflammation in the treated MSA mice. While fingolimod itself has not yet been trialed in MSA patients, these results point to the possibility of repurposing immunomodulators from multiple sclerosis to MSA, targeting both inflammation and providing trophic support.

### Targeting mitochondrial dysfunction, oxidative stress, and excitotoxicity

MSA involves not only protein aggregates and inflammation, but also significant cellular stress, including mitochondrial dysfunction and glutamate excitotoxicity [1, 52]. Mitochondrial impairment and resultant oxidative damage are thought to contribute significantly to neurodegeneration in MSA [53]. Genetic and biochemical evidence links Coenzyme Q10 (CoQ10) – a vital mitochondrial cofactor – to MSA. Notably, variants of the *COQ2* gene, which encodes an enzyme in CoQ10 biosynthesis, have been identified in familial MSA cases, and a specific variant (V393A) is associated with sporadic MSA in East Asian cohorts [54, 55]. Furthermore, MSA patients have reduced levels of CoQ10 in their tissues [56, 57]. CoQ10 (also known as ubiquinone) plays a dual role as an essential electron carrier in the mitochondrial respiratory chain and a potent lipid-soluble antioxidant. Deficiency of CoQ10 can lead to inefficient oxidative phosphorylation and excessive reactive oxygen species, exacerbating neuronal injury [58]. In MSA, the combination of CoQ10 deficiency and a high oxidative stress burden provides a strong rationale for CoQ10 supplementation as a neuroprotective strategy.

Additionally, excitotoxic injury due to glutamate dysregulation may contribute to neuronal death [59]. Therefore, drugs that support mitochondrial function or reduce excitotoxicity have been repurposed from other neurodegenerative conditions. Table 3 summarizes the therapeutic agents targeting this pathway, detailing their specific mechanisms, evidence from preclinical studies, results from clinical trials, and current developmental status.

**Table 3:** Summary of therapies targeting mitochondrial dysfunction, oxidative stress, and excitotoxicity in multiple system atrophy

Drug/Therapy	Original indication	Mechanism of action	Preclinical evidence	Clinical trial outcome	Current status
Ubiquinol	Nutraceutical/Dietary supplement for mitochondrial support; used in mitochondrial disorders, cardiovascular disease, and as a general antioxidant supplement	Acts as a cofactor in the mitochondrial electron transport chain (ETC), supporting ATP production; scavenges reactive oxygen species as a lipid-soluble antioxidant. Reduces oxidative stress and improves mitochondrial bioenergetics	In MSA model systems and *COQ2* mutation carriers, ubiquinol supplementation improved mitochondrial function. A long-term case study reported clinical stability and improved oxidative metabolism with high-dose ubiquinol in familial MSA	A 48-week, Phase 2 randomized placebo-controlled trial (MSA-01, 2023) demonstrated a significantly slower progression in UMSARS Part 2 scores with ubiquinol 1500 mg/day vs. placebo. Secondary endpoints (e.g., Barthel Index, SARA) also favored treatment. No serious adverse events reported	Supported by positive Phase 2 trial results. Considered a promising disease-modifying candidate. Phase 3 trial is anticipated; currently off-label use in MSA with high-dose administration under clinical supervision
Riluzole	Glutamate-release inhibitor (ALS therapy)	Reduces glutamatergic excitotoxicity: blocks presynaptic sodium channels (↓glutamate release); mild NMDA receptor antagonism	Yes – MSA double-lesion rats: improved motor function, preserved striatal neurons	Large RCT (NNIPPS, in MSA & PSP, up to 3 years): no extension of survival or slowed progression in MSA; small MSA-only trial also no benefit; well tolerated	No disease-modifying effect in MSA; no further development for this indication
Inosine (uric acid enhancer)	Nutritional supplement (purine precursor) used experimentally	Metabolic precursor that raises serum uric acid (antioxidant and peroxynitrite scavenger); higher urate may reduce α-synuclein propagation	Yes – PD models: increases uric acid, reduces α-syn spread and neurodegeneration; higher serum urate correlates with slower MSA progression (observational)	Phase II trial (IMPROVE-MSA, 24 weeks): inosine monophosphate safely raised serum uric acid (~ 4.6 to 7.0 mg/dL) vs. placebo; well tolerated (few cases of asymptomatic hyperuricemia); efficacy not assessed in short trial	Investigational – safe strategy to boost antioxidant levels; effect on disease progression unproven (further trials needed)
Rasagiline	MAO-B inhibitor (Parkinson’s disease adjunct)	Inhibits monoamine oxidase B (↑dopamine levels); anti-apoptotic and mitochondrial protective effects	Yes – MSA transgenic (oligodendroglial α-syn) mice: improved motor performance, ↓glial inclusions, ↑neuronal survival	Phase III trial in MSA-P (48 weeks): no significant difference in UMSARS progression vs. placebo; no clinical benefit; well tolerated (watch for orthostatic hypotension)	No efficacy in MSA; not pursued as disease-modifying therapy
Safinamide	MAO-B inhibitor + glutamate modulator (Parkinson’s adjunct)	Dual mechanism: inhibits MAO-B (dopamine-enhancing) and blocks voltage-gated Na^+^ channels (reduces glutamate release)	No specific MSA preclinical studies (mechanism-based rationale from PD)	Phase II trial in MSA: generally safe/tolerable; no significant improvement in motor function or progression observed	Safe but no evident benefit; no further trials reported in MSA

MSA, multiple system atrophy; ETC, electron transport chain; ROS, reactive oxygen species; ATP, adenosine triphosphate; *COQ2*, coenzyme Q2 gene; UMSARS, Unified MSA Rating Scale; ALS, amyotrophic lateral sclerosis; NMDA, N-methyl-D-aspartate; MAO-B, monoamine oxidase B

#### Ubiquinol

Ubiquinol, the reduced, more bioavailable form of CoQ10, is of particular interest. By restoring mitochondrial electron transport and directly scavenging free radicals, ubiquinol may bolster cellular energy production and prevent oxidative damage to neurons and glia. MSA and other synucleinopathies like PD have long been hypothesized to benefit from mitochondrial support by CoQ10, given evidence of oxidative stress and impaired energy metabolism in these disorders [60]. However, large trials in PD have yielded equivocal or negative results for CoQ10 [61, 62], possibly due to late intervention or insufficient target engagement. In MSA, the findings of *COQ2* mutations and CoQ10 deficiency have prompted investigations of high-dose ubiquinol as a disease-modifying therapy.

Mitsui et al. reported a case of familial MSA with compound *COQ2* mutations, who received high-dose ubiquinol (1200–1500 mg/day) over 3 years [63]. The treatment was well-tolerated and notably, the patient’s mitochondrial oxidative metabolism improved, with clinical rating scores remaining stable throughout the 3-year period. This long-term single-case study hinted at disease stabilization, supporting the concept that augmenting CoQ10 levels might modify the trajectory of MSA, especially in genetically predisposed cases.

Building on this rationale, a multicenter, randomized, double-blind, placebo-controlled Phase 2 trial in Japan tested oral high-dose ubiquinol (titrated to 1500 mg/day once daily) over 48 weeks, with a 4-week washout period [64]. In total, 139 patients were randomized (69 ubiquinol, 70 placebo), and the full analysis set included 61 vs. 68. The primary endpoint—change in UMSARS-II from baseline to week 48—favored ubiquinol (least squares mean change 5.4 in the treatment group vs. 7.1 in the placebo group; difference − 1.7, 95% CI − 3.2 to − 0.2; *P* = 0.023), thus meeting the primary efficacy outcome. Secondary outcomes were directionally consistent (e.g., Barthel Index difference + 5.3; Scale for the Assessment and Rating of Ataxia − 1.4; 10-m walk − 24.1 s), though they did not reach nominal statistical significance at week 48; UMSARS-I changes were similar (4.7 in the treatment group vs. 4.9 in the placebo group; *P* = 0.864). Prespecified subgroups suggested a numerically larger effect in MSA-P (− 4.2) than in MSA-C (− 1.0), without significant interactions, and results appeared irrespective of *COQ2* (V393A) status (carriers − 1.8; non-carriers − 1.6). After 4-week washout (week 52), UMSARS II changed minimally (0.4 in the treatment group vs. 0.5 in the placebo group), supporting a disease-modifying rather than a purely symptomatic effect. The overall and drug-related AE rates were comparable (potentially related AEs 23.8% in the treatment group vs. 30.9% in the placebo group), but serious AEs occurred more often with ubiquinol (31.7% in the treatment group vs. 17.6% in the placebo group). The investigators judged two gastrointestinal serious AEs to be possibly related and four deaths in the ubiquinol arm unrelated to the study drug. This marks a breakthrough, as ubiquinol is the first agent in a placebo-controlled trial to show a significant disease-modifying signal in MSA. By targeting mitochondrial dysfunction and oxidative injury, ubiquinol supplementation may help preserve neurons and oligodendroglia from energetic deficits in MSA.

#### Riluzole

Riluzole is a glutamate-release inhibitor and sodium channel blocker that is approved by the FDA as a disease-modifying treatment for amyotrophic lateral sclerosis (ALS) [65]. In ALS, riluzole prolongs survival modestly, presumably by reducing glutamate-mediated excitotoxicity to motor neurons. Given its neuroprotective profile, riluzole has been tested in atypical parkinsonian disorders including MSA. Mechanistically, riluzole reduces neuronal excitability by blocking presynaptic sodium channels, thereby reducing glutamate release. It also directly antagonizes NMDA receptors and voltage-gated calcium channels [66]. This leads to lower extracellular glutamate and protection against overexcitation of neurons. In a double-lesion rat model of MSA, riluzole treatment improved motor deficits and attenuated the loss of striatal neurons [67]. These preclinical data support a possible disease-modifying effect.

To evaluate this, a phase-III, randomized, double-blind, placebo-controlled study, the large international NNIPPS trial, tested oral riluzole (flexible dosing 50–200 mg/day) in Parkinson-plus syndromes and followed patients for up to 36 months (median 1095 days) [68]. In total, 767 patients were randomized (MSA = 398; PS*P* = 362), with 760 entering the intention-to-treat analysis. The primary endpoint was survival; riluzole did not prolong survival in MSA (3-year Kaplan–Meier estimates 0.53 riluzole vs. 0.58 placebo; log-rank *P* = 0.48) or in the overall cohort (stratified log-rank *P* = 0.42). Functional progression—assessed semi-annually with Schwab and England activities of daily living (ADL) scale, Hoehn & Yahr, CGI-severity/dysautonomia, and the Short Motor Disability Scale—also did not differ between riluzole and placebo. Gastrointestinal AEs were more frequent with riluzole. Also, a smaller, placebo-controlled cross-over trial specific to MSA tested oral riluzole 100 mg twice daily (200 mg/day) for two 4-week treatment periods separated by a 4-week washout (*n* = 10)​ [69]. The primary outcomes were short-term symptomatic effects on UPDRS-II (ADL) and UPDRS-III (motor) (UMSARS was not used). No motor benefit emerged over the short treatment periods: the riluzole–placebo differences in change were UPDRS-II − 0.1 (95% CI − 1.1 to 0.9), UPDRS-III − 0.2 (− 2.0 to 1.6), and combined UPDRS-II + III − 0.2 (− 2.4 to 2.0), none reaching significance. Riluzole was generally well tolerated, and all patients completed the trial. Taken together, unlike in ALS, riluzole showed no disease-modifying or symptomatic effect in MSA. The drug was generally well tolerated in these patients. The failure of riluzole suggests that excitotoxicity might not be the dominant driver in MSA, or that by the time of diagnosis, blocking glutamate is too late. Riluzole is no longer considered a promising therapy for MSA based on these trial results.

#### Uric acid and inosine

Uric acid, a byproduct from purine metabolism with antioxidant properties, acts as a scavenger of reactive oxygen species, a peroxynitrite scavenger, and an iron chelator. These properties make it a candidate for mitigating oxidative stress, a key factor in MSA pathogenesis, which involves mitochondrial dysfunction and neuronal loss. In addition to the antioxidative role of uric acid, our previous study found that uric acid inhibits cell-to-cell transmission of α-synuclein in PD models by blocking endocytosis pathways, specifically dynamin-mediated and clathrin-mediated endocytosis [70]. In α-synuclein-inoculated mice, elevating the uric acid level reduced α-synuclein propagation, decreased levels of phosphorylated α-synuclein, and improved motor function, without affecting autophagy or microglial activation. Although this mechanism was investigated in PD models, we found that uric acid also suppresses cell-to-cell transmission of α-synuclein. These findings indicate that the function of uric acid extends beyond its antioxidant effects, influencing a core pathogenic process in MSA—oligodendroglial α-synuclein propagation. Our another clinical study found that higher serum levels of uric acid correlate with slower disease progression in MSA [71]. We also found an association between serum urate levels and white matter integrity in MSA patients, which suggested that the protective effect of urate on white matter integrity is linked to reduced disease severity [72].

The IMPROVE-MSA trial evaluated inosine 5'-monophosphate (IMP) in a 24-week randomized, double-blind, placebo-controlled study involving 55 MSA patients (30 assigned to IMP and 25 to placebo) [73]. IMP, a purine nucleoside precursor, has the ability to enhance uric acid levels. The trial demonstrated that IMP treatment safely and effectively increased serum uric acid from 4.57 mg/dL to 6.96 mg/dL, with no significant increase in AEs compared to placebo. The UMSARS changes over 24 weeks did not differ between arms (IMP vs. placebo: total + 1.9 vs. + 2.5; Part I + 0.9 vs. + 1.5; Part II − 0.5 vs. + 0.4; all nonsignificant), while cognition showed a signal favoring IMP (MoCA improved with IMP vs. placebo, *P* < 0.001; MMSE trend *P* = 0.09). Three IMP recipients developed asymptomatic hyperuricemia (UA ≥ 9 mg/dL), all managed by dose reduction/temporary hold; no gout occurred. Serious AEs were similar (20.0% in the treatment group vs. 16.0% in the placebo group), with deaths of 1 vs. 2, none attributed to the study drug. This approach leverages the role of inosine in purine metabolism to boost the antioxidant capacity, potentially slowing MSA progression by enhancing the neuroprotective effects of uric acid. However, a further trial with long-term follow-up is required to examine whether UA elevation will have a disease-modifying effect in MSA.

#### Rasagiline and safinamide

Rasagiline is a selective monoamine oxidase-B (MAO-B) inhibitor approved for PD as an adjunct to levodopa. Beyond its symptomatic effect, a delayed-start trial (ADAGIO) has suggested a possible disease-modifying effect of rasagiline 1 mg in PD, though interpretations were debated [74]. These findings prompted investigation of rasagiline in MSA, especially MSA-P. In a transgenic MSA mouse model with oligodendroglial α-synuclein [75], rasagiline treatment resulted in improved motor performance, a reduction in the density of GCIs, and greater neuronal survival compared to controls. This indicated that rasagiline could penetrate the brain and exert neuroprotective effects in the context of synuclein pathology. In a multicenter randomized double-blind trial conducted at 40 sites across 12 countries, 174 patients with possible or probable MSA-P were assigned to receive either rasagiline 1 mg/day (*n* = 84) or placebo (*n* = 90) for 48 weeks. Progression in UMSARS I + II was similar between groups, with an adjusted mean increase of 7.2 units in the rasagiline group versus 7.8 units in the placebo group (treatment difference –0.60; 95% CI –3.68 to 2.47; *P* = 0.70) [76]. No meaningful slowing of motor or autonomic decline was observed. Rasagiline was well tolerated in MSA (no major safety issues, aside from its known risk of hypotension which is relevant since MSA patients often have orthostatic hypotension). The lack of efficacy suggests that even though rasagiline can modulate mitochondrial dysfunction and has anti-apoptotic properties, its administration alone is insufficient to impact the aggressive disease course of MSA.

Safinamide is another MAO-B inhibitor originally developed for PD, with a dual mechanism [77]. It inhibits MAO-B and also modulates glutamate release by blocking voltage-dependent sodium channels at higher doses, thus both enhancing dopaminergic tone and potentially reducing the excitotoxic glutamate transmission [78]. Therefore, safinamide was considered for MSA-P as well. A small Phase II trial was conducted to primarily assess AEs and exploratory efficacy endpoints. The trial (NCT03753763) results showed that safinamide is generally safe in MSA patients, with no unexpected AEs. However, it did not produce a significant improvement in motor function or slowing of progression over the study period. These results have not yet been published in a peer-reviewed journal.

### Enhancing neurotrophic support and neuroprotection

Loss of neurotrophic support is thought to contribute to the degeneration seen in MSA. Oligodendroglial and neuronal cell death may be exacerbated by reduced levels of growth factors like BDNF and GDNF in affected brain regions [79]. Therefore, strategies to boost these survival-promoting factors or mimic their effects are of great interest. Several repurposed drugs and biologics fall into this category, aiming to create a more neuroprotective milieu in the MSA brain. Table 4 provides an overview of drugs focused on this pathway, including their mechanisms, preclinical findings, clinical trial results, and current development status.

**Table 4:** Summary of therapies enhancing neurotrophic support and neuroprotection in multiple system atrophy

Drug/Therapy	Original indication	Mechanism of action	Preclinical evidence	Clinical trial outcome	Current status
Fluoxetine (SSRI antidepressant)	Antidepressant (major depressive disorder)	Selective serotonin reuptake inhibitor; increases BDNF/GDNF and other neurotrophic factors; anti-inflammatory effects in CNS	Yes – MSA mice: chronic fluoxetine increases BDNF & GDNF, decreases pro-inflammatory cytokines; SSRIs in general elevate neurotrophic support	Phase II placebo-controlled trial: no significant difference in primary outcome (UMSARS); slight trend toward slower motor decline and improved mood in treated group (not statistically significant)	Not proven disease-modifying; used for symptom management (depression); further study of higher doses/other SSRIs ongoing
Intranasal Insulin	Hormone (diabetes) – repurposed via intranasal delivery for CNS	Insulin delivered intranasally to target brain insulin pathways; promotes neuronal survival and metabolism; may reduce α-syn accumulation via improved insulin sensitivity	Anecdotal – MSA model: evidence of insulin resistance; pilot in PD & one MSA case: the MSA patient on intranasal insulin had no progression over 4 weeks (versus expected decline)	No controlled trials in MSA yet (only single-patient observation); in PD, intranasal insulin showed cognitive/motor benefits in small studies	Experimental therapy – promising, rationale but unproven; further clinical trials needed to assess efficacy in MSA
Growth Hormone (GH)	Hormone therapy for GH deficiency	Stimulates IGF-1 production and downstream neurotrophic pathways; supports neuronal growth and survival	Limited – based on low IGF-1 observed in MSA models; no specific animal trial of GH in MSA	Small 12-month RCT of GH in MSA: no significant difference vs. placebo (underpowered); feasible but no clear efficacy	Investigational – not shown effective; focus shifted to other ways of enhancing IGF-1 (e.g. GLP-1 agonists)
Exendin-4 (exenatide)	GLP-1 receptor agonist (type 2 diabetes medication)	Activates GLP-1 receptors: improves insulin signaling, reduces inflammation, and enhances mitochondrial function; neurotrophic and neuroprotective effects	Yes – MSA mice: decreased α-synuclein accumulation, preserved dopaminergic neurons (no marked motor improvement in model); PD models: neuroprotective, improved motor in some trials	Open-label phase II trial in early MSA (48 weeks): slower worsening of UMSARS I + II compared with standard care, but no differences in objective secondary outcomes or biomarkers (NfL, CSF α-synuclein oligomers, MRI/VBM); interpretation limited by open-label design, with no clear evidence of disease modification	In clinical trial – considered a promising candidate; awaiting trial outcomes to determine efficacy
Benztropine	Anticholinergic agent for parkinsonism/dystonia	Central antimuscarinic; additionally promotes OPC-to-oligodendrocyte differentiation and remyelination	Yes – MSA-specific model: In α-synuclein-overexpressing oligodendrocyte cultures and in MBP29-hα-syn transgenic MSA mice, benztropine restored myelin protein expression, enhanced remyelination, and prevented neuronal loss	None in MSA	Investigational for myelin/oligodendroglial repair in α-synucleinopathies
Sobetirome (TRβ-selective thyromimetic)	Dyslipidemia/thyroid-axis modulation (repurposed for CNS myelin repair)	Brain-penetrant TRβ agonist; promotes oligodendrocyte maturation and myelination, leading to potential restoration of oligodendroglial function	Yes – In vitro MSA model (MBP29 α-syn-oligodendrocyte): sobetirome rescued myelin protein expression and increased myelinated segments	None in MSA	Experimental/early stage; biologically compelling for MSA’s oligodendroglial pathology; needs in vivo MSA data and clinical translation

MSA, multiple system atrophy; SSRI, selective serotonin reuptake inhibitor; BDNF, brain-derived neurotrophic factor; GDNF, glial cell line-derived neurotrophic factor; IGF-1, insulin-like growth factor 1; GLP-1, glucagon-like peptide-1; PD, Parkinson’s disease; OPC, oligodendrocyte precursor cell; TRβ, thyroid hormone receptor beta; CNS, central nervous system

#### Fluoxetine and other SSRIs

Fluoxetine, a widely used antidepressant belonging to SSRIs, is an example of an unexpected repurposing candidate for MSA. Aside from treating depression, fluoxetine was hypothesized to have direct neuroprotective effects. In vitro and animal studies have indicated that SSRIs can increase the expression of neurotrophic molecules such as BDNF and GDNF in the brain​ [80, 81]. Specifically, in a transgenic MSA mouse model, chronic fluoxetine treatment led to elevated levels of GDNF and BDNF, and suppressed production of pro-inflammatory cytokines in the central nervous system (CNS) [81, 82].

The randomized, double-blind, placebo-controlled Phase II MSA-FLUO trial (NCT01146548) tested oral fluoxetine (20 mg/day for 6 weeks → 40 mg/day to week 24) over 24 weeks. A total of 81 patients were randomized (40 fluoxetine, 41 placebo). The primary endpoint—change from baseline to week 12 in total UMSARS (Parts I + II)—showed no significant difference (adjusted treatment effect − 2.13 points; 95% CI − 4.55 to 0.29; *P* = 0.08). By group, UMSARS I + II increased from 40.3 to 41.6 with fluoxetine (+ 1.3) vs. 40.2 to 43.3 with placebo (+ 3.1). The UMSARS Part II worsened less on fluoxetine (21.1 → 21.4, + 0.3) than placebo (21.5 → 23.0, + 1.5; adjusted effect − 1.41, 95% CI − 2.84 to 0.03; *P* = 0.05), while Part I changes were similar (19.2 → 20.2, + 1.0 vs. 18.7 → 20.3, + 1.6; *P* = 0.34). Among the secondary outcomes, the MSA-QoL emotional/social dimension favored fluoxetine (− 6.99, 95% CI − 13.40 to − 0.56; *P* = 0.03), with no difference in Scales for Outcomes in Parkinson’s Disease–Autonomic or BDI [83]. Safety was consistent with known SSRI effects: serious AEs were 28% vs. 17% (fluoxetine vs. placebo), dose interruption/down-titration 28% vs. 11%, and deaths 3 vs. 2, none treatment-related. After this trial, an independent long-term observational study of MSA patients (retrospective analysis of over 600 cases) found that those who had ever used SSRIs did not live longer than those who never did, nor was there a slower disability accrual [84]. Overall, the evidence does not support a major disease-modifying effect of fluoxetine or other SSRIs in MSA.

#### Insulin and the insulin-like growth factor-1 (IGF-1) pathway

Intriguingly, recent evidence suggests that the insulin signaling pathways are altered in MSA, leading to the idea of repurposing metabolic hormones for therapy. Insulin and IGF-1 play important roles in the normal brain by regulating neuronal metabolism, growth, and synaptic plasticity. They are also implicated in neurodegenerative diseases [85, 86]. In MSA, a study found elevated peripheral insulin levels (suggesting insulin resistance) and reduced IGF-1 levels in the brains of MSA model mice [87–89]. These findings mirror the insulin resistance state and raise the question of whether improving insulin signaling in the brain could be neuroprotective. Intranasal insulin is a method to deliver insulin to the CNS while bypassing the peripheral circulation. A pilot trial in 2019 treated patients with PD and one patient with MSA used daily intranasal insulin (40 IU human regular insulin once daily via ViaNase) for 4 weeks [90]. In patients with PD, intranasal insulin improved verbal fluency and motor scores without systemic side effects. Remarkably, the single MSA patient remained clinically stable over 4 weeks with a slight trend to improvement on some measures (HY 2.5 → 2.5; UPDRS-III 36 → 34; UPDRS-II 18 → 20; UPDRS-I 9 → 8; Fluency, Attention, and Speed test 44 → 46). UMSARS was not assessed in this trial. While this is an anecdotal result (*N* = 1 for MSA), it hinted that insulin might have a stabilizing effect.

Along with the neurotrophic and metabolic enhancement, insulin may indirectly reduce α-synuclein accumulation, as seen in the study by Bassil et al., where improving insulin sensitivity with exendin-4 reduced the α-synuclein burden in MSA mice [87]. As of now, insulin remains an experimental, off-label approach in MSA, with some encouraging early data. If ongoing studies confirm cognitive or motor benefits, it could become a low-cost repurposed therapy used alongside other treatments.

It is worth noting that another agent affecting the IGF-1 pathway, growth hormone (GH), was also tried in MSA. An international, multicenter, randomized, double-blind, placebo-controlled pilot trial tested subcutaneous recombinant human GH (r-hGH) for 12 months in MSA [91]. Patients received 1.0 mg every other day for 6 months, then alternating daily 1.0 mg and 0.5 mg for a further 6 months. Forty-three patients were randomized (r-hGH 22; placebo 21). The trial’s primary efficacy measures were UPDRS total and cardiovascular reflex autonomic testing (HRV during deep breathing; MAP% on head-up tilt); UMSARS total was secondary. r-hGH did not show a statistically significant advantage over placebo: UMSARS total worsened by + 6.6 ± 8.6 vs. + 9.9 ± 6.5 at 6 months, and + 10.6 ± 11.8 vs. + 17.1 ± 8.6 at 12 months (r-hGH vs. placebo; 95% CIs overlapped); UPDRS and autonomic measures also showed only non-significant trends favoring r-hGH. Safety was broadly similar between groups (serious AEs, 8 in r-hGH vs. 10 in placebo group); four discontinuations due to AEs (3 in r-hGH—including one hyperglycemia judged probably related, and 1 in placebo). Overall, feasibility constraints and the small sample size limited definitive conclusions. The GH/IGF-1 axis remains a potential target, but attention has shifted more to glucagon-like peptide-1 (GLP-1) analogs such as exendin-4 which can be given as a simple injection and have direct neuroprotective evidence.

#### Exendin-4

Exendin-4 (exenatide) is a GLP-1 analog originally developed for type 2 diabetes. Beyond the glucose-lowering effects, GLP-1 analogs have demonstrated neuroprotective properties in multiple models of neurodegeneration​ [87]. They cross the blood–brain barrier and act on neuronal GLP-1 receptors, which can enhance insulin signaling, reduce inflammation, and improve mitochondrial function. In the context of MSA, exendin-4 has been tested in the same transgenic mouse model mentioned earlier (transgenic PLP-α-synuclein mice). Bassil et al. reported that exendin-4 treatment in these MSA mice resulted in reduced α-synuclein accumulation in the striatum and improved survival of nigral dopaminergic neurons. Interestingly, despite these neuropathological improvements, the study noted that exendin-4 did not significantly change the motor phenotype of the mice during the treatment period [87]. This suggests that while the drug was biologically active, it did not fully translate to functional gains in the mice—perhaps due to the aggressive nature of the model or the endpoints used.

Nonetheless, given the strong rationale and safety record in diabetes, a clinical trial was initiated to explore exendin-4 in MSA patients. A UK-based Phase II trial (NCT04431713) testing exenatide in patients with early MSA has recently been completed and published [92]. A total of 50 participants were enrolled and randomized 1:1 in an open-label design to receive weekly subcutaneous exenatide 2 mg injection or standard care alone for 48 weeks, followed by a 48-week washout period. The primary outcome was the change in the combined UMSARS parts I and II score at 48 weeks. At week 48, the exenatide group showed a smaller worsening in UMSARS I + II scores compared with controls (mean change + 6.1 in the treatment group vs. + 13.3 points in the control group), corresponding to an adjusted mean difference of − 7.4 points (*P* < 0.001). However, biomarker analyses including serum NfL, CSF α-synuclein oligomer burden, and MRI-based measures such as voxel-based morphometry did not differ between treatment groups. Taken together, while exenatide was associated with improvements in participant-reported symptoms and clinician-rated global severity, the absence of corresponding changes in objective biomarkers raises uncertainty regarding the disease-modifying effects. Given the open-label design, these findings may reflect symptomatic benefit, expectancy effects, or observer bias. Placebo-controlled trials with blinded outcome assessment and biomarker-based target engagement will therefore be required to determine whether GLP-1 analogs exert true disease-modifying effects in MSA.

#### Future candidates associated with remyelination

Given that oligodendroglial degeneration and consequent myelin loss are central pathological features of MSA [93], strategies that promote remyelination represent a promising future direction. Several repurposed drugs originally developed for other neurological or systemic indications have shown myelin-restorative potential in preclinical studies.

Benztropine, a centrally acting muscarinic antagonist historically used to treat parkinsonian tremor, has been shown to enhance oligodendrocyte precursor cell differentiation and stimulate myelin repair in demyelinating models. Notably, one study demonstrated that benztropine restored myelination and preserved neuronal integrity in a transgenic MSA mouse model overexpressing α-synuclein in oligodendrocytes [94], possibly suggesting that pharmacological enhancement of oligodendrocyte differentiation could represent a viable disease-modifying strategy. Another compound of interest is sobetirome, a thyroid-hormone receptor-β agonist that promotes myelin protein expression and oligodendrocyte maturation. A recent study reported that sobetirome counteracted the α-synuclein-mediated demyelination in oligodendroglial cultures, supporting the rationale for targeting thyroid-hormone signaling to restore myelin integrity [95]. Beyond these, drugs currently being tested in multiple sclerosis, such as clemastine (a first-generation antihistamine with antimuscarinic properties) and PIPE-307 (a selective M1 muscarinic receptor antagonist), have demonstrated myelin-restorative effects and may warrant exploration in MSA. While none of these agents have yet been evaluated clinically in MSA, their mechanisms—enhancing oligodendrocyte differentiation, thyroid-hormone-mediated myelin synthesis, and muscarinic pathway modulation—directly address a major pathological component of the disease. Integrating such glia-targeted approaches into future MSA research could broaden the scope of neuroprotective strategies beyond neuronal survival and toward restoration of myelin and network integrity.

## Discussion

A detailed summary of completed repurposing trials in MSA is presented in Table 5, including key trial characteristics such as NCT identifiers, enrollment size, endpoints, and outcome metrics. Despite significant progress in the exploration of drug repurposing strategies for MSA, no treatment has yet demonstrated robust disease-modifying efficacy. Several key insights emerge when examining the collective experience from past clinical trials.

**Table 5:** Comparative overview of completed clinical trials evaluating repurposed drugs in multiple system atrophy

Drug/NCT No.	Trial name (Acronym)	Trial phase/duration	*N* (Drug/Placebo)	Dose	Primary endpoint/main result	References
Sirolimus/NCT03589976	A Futility Trial of Sirolimus in MSA	Phase II RCT, 48 weeks	35 (MSA-P 18; MSA-C 17)/12 (MSA-P 7; MSA-C 5)	2–6 mg/day	Δ UMSARS total – no difference (*P* = 0.65)	[14]
Rifampicin/NCT01287221	Study of Rifampicin in Multiple System Atrophy	Phase III RCT, 12 months	50 (MSA-P 19; MSA-C 31)/50 (MSA-P 22; MSA-C 28)	300 mg BID	Δ UMSARS-I rate – no benefit (*P* = 0.82)	[17]
Lithium/NCT00997672	Lithium in MSA	Phase II RCT, 48 weeks	4/5 (All enrolled patients were MSA-C)	150–1500 mg/day	Safety/tolerability – trial stopped early	[24]
EGCG/NCT02008721	Progression Rate of MSA Under EGCG Supplementation as Anti-Aggregation-Approach (PROMESA)	Phase II/III RCT, 52 weeks (4-week wash-out period)	47 (MSA-P 25; MSA-C 22)/45 (MSA-P 24; MSA-C 21)	≤ 1200 mg/day	Δ UMSARS-II – NS (*P* = 0.51)	[30]
Minocycline/NCT00146809	Study About Efficacy and Safety to Treat MSA (MEMSA)	Phase II RCT, 48 weeks	32/31 (All enrolled patients were MSA-P)	200 mg/day	Δ UMSARS-II – NS (*P* = 0.18)	[46]
IVIg/NCT00750867	Treatment of Multiple System Atrophy Using Intravenous IVIgs	Open-label pilot, 6 months	9 (MSA-P 2; MSA-C 5; unknown 2 [discontinuation])	0.4 g/kg monthly	Δ UMSARS I/II – improved (*P* < 0.05)	[48]
Ubiquinol/UMIN000031771	High-Dose Ubiquinol Supplementation in MSA	Phase II RCT, 48 weeks	69 (MSA-P 13; MSA-C 48)/70 (MSA-P 17; MSA-C 51)	1500 mg/day	Δ UMSARS-II – improved (*P* = 0.023)	[64]
Riluzole/NCT00211224	Neuroprotection and Natural History in Parkinson's Plus Syndromes (NNIPPS)	Phase III RCT, ≤ 3 years	398 (MSA subset, no subtype information)	50–200 mg/day	Survival – no difference (*P* = 0.48)	[68]
Rasagiline/NCT00977665	Clinical Trial to Assess Efficacy, Safety, and Tolerability of Rasagiline Mesylate 1 mg in Patients With MSA of the Parkinsonian Subtype (MSA-P)	Phase II RCT, 48 weeks	84/90 (all enrolled patients were MSA-P)	1 mg/day	Δ UMSARS I + II – NS (*P* = 0.70)	[76]
Fluoxetine/NCT01146548	Fluoxetine in Multiple System Atrophy Patients (MSA-Fluox)	Phase II RCT, 24 weeks	40 (MSA-P 24; MSA-C 16)/41 (MSA-P 20; MSA-C 21)	20–40 mg/day	Δ UMSARS I + II – trend (*P* = 0.08)	[83]
Growth Hormone/N/A	Safety and tolerability of growth hormone therapy in MSA	Phase IIa RCT, 12 months	22 (MSA-P 14; MSA-C 8)/21 (MSA-P 12; MSA-C 9)	1 mg SC qod	Δ UMSARS total – NS	[91]
Inosine/NCT03403309	Inosine 5′-Monophosphate to Raise Serum Uric Acid Level in Patients With MSA (IMPROVE-MSA)	Phase II RCT, 24 weeks	30 (MSA-P 5; MSA-C 22; mixed 3)/25 (MSA-P 6; MSA-C 17; mixed 2)	Titrated to UA 6–8 mg/dL	↑ Serum UA – safe, no efficacy signal	[73]
Exendin-4 (Exenatide)/NCT04431713	Exenatide Once-weekly as a Treatment for MSA	Phase II randomized open-label, 48 weeks (48-week washout period)	25 (MSA-P 14; MSA-C 11)/25 (MSA-P 11; MSA-C 14)	2 mg SC weekly	Δ UMSARS I + II – smaller worsening vs. standard care at 48 weeks (*P* < 0.001); no differences in objective biomarkers (NfL, CSF α-syn oligomers, MRI/VBM)	[92]

EGCG, epigallocatechin-3-gallate; GLP-1, glucagon-like peptide-1; IVIg, intravenous immunoglobulin; MSA, multiple system atrophy; MRI, magnetic resonance imaging; *N*, number of participants; NCT, National Clinical Trial identifier; NS, non-significant; N/A, not available; PD, Parkinson’s disease; RCT, randomized controlled trial; SC, subcutaneous; UA, uric acid; UMSARS, Unified Multiple System Atrophy Rating Scale; VBM, voxel-based morphometry

First, the timing of intervention appears critical. Many negative studies enrolled patients at relatively advanced stages, when neuronal and oligodendroglial pathology was already extensive. For example, rifampicin, advanced directly into a 12-month Phase III trial without an intermediate Phase II, failed to slow clinical decline despite promising preclinical results [17]. Similarly, minocycline reduced microglial activation on PET imaging, confirming biological target engagement, but showed no clinical benefit in probable MSA patients with advanced disease [46]. These findings suggest that interventions initiated earlier in the disease course—perhaps even at the “possible prodromal MSA” stage—are more likely to demonstrate clinical efficacy [96]. In rapidly progressive disorders like MSA, the window between cohort identification and actual trial initiation is narrow. Even in well-characterized, trial-ready cohorts, patients may experience functional decline during the study start-up phase, rendering them ineligible for participation. As a result, this limits the feasibility and utility of conventional cohort-ready paradigms in MSA. In this regard, establishing longitudinal cohorts of individuals at prodromal or pre-symptomatic stages—such as those with isolated autonomic failure, REM sleep behavior disorder, or subtle cerebellar signs—may provide a more sustainable pathway. Future MSA drug development efforts should thus invest in building and tracking such prodromal cohorts, alongside refining criteria to predict phenoconversion with confidence.

A second consistent barrier has been the structural limitations of past trial designs. Many studies were of relatively short duration (6–12 months), which may be insufficient to capture disease-modifying effects in a rapidly progressive disorder such as MSA. Sample sizes have also been limited, reflecting the rarity of the condition, and necessitating multicenter or international collaborations that introduce logistical complexity. Furthermore, the lack of reliable biomarkers has hindered both early diagnosis and the objective measurement of therapeutic impact. Although several candidate biomarkers—such as serum and CSF NfL, α-synuclein seed amplification assays such as RT-QuIC (real-time quaking-induced conversion) or PMCA (protein misfolding cyclic amplification), and quantitative MRI metrics of putaminal atrophy—have shown potential, none are yet standardized or validated for regulatory use in MSA. This has important consequences: without sensitive diagnostic and prognostic markers, trials cannot easily identify early-stage or rapidly progressing patients, nor can they confirm target engagement or biological response. In addition, biomarker variability across centers and lack of longitudinal datasets impede comparisons and meta-analysis, further complicating trial design. Moreover, the rarity of MSA and its rapid clinical progression make it inherently difficult to recruit an adequate sample size and maintain longitudinal follow-up. On the other hand, these same features argue that MSA is intrinsically a disease that requires larger and more carefully powered trials. Substantial heterogeneity exists both between MSA-P and MSA-C phenotypes and within individual subtypes, with disease progression trajectories varying widely. While the overall median survival is typically reported at approximately 7–10 years from symptom onset [6], pathologically confirmed cases have demonstrated survival exceeding 15–20 years in so-called benign variants, whereas aggressive phenotypes with disease duration of less than 3 years have also been described [97–99]. As summarized in Table 6, most completed trials have relied predominantly on global clinical scales such as UMSARS as primary endpoints, with limited incorporation of objective biomarkers or target-engagement measures. While UMSARS provides a standardized assessment of overall disease severity, its composite structure may lack sensitivity to detect domain-specific or mechanism-driven therapeutic effects in a biologically heterogeneous disorder such as MSA [100]. Integrating multimodal biomarkers—combining fluid, imaging, and digital (wearable-based) measures—may allow earlier stratification and reduce required sample sizes by lowering within-group variability. Collectively, these obstacles highlight the need for smarter trial designs that address patient selection, trial length, and mechanistic endpoints more effectively. In this context, glial biomarkers such as glial fibrillary acidic protein and myelin-related markers, including myelin basic protein, merit increased attention. Given that oligodendroglial dysfunction and demyelination are defining pathological features of MSA, these markers may offer greater disease specificity and improved sensitivity to therapeutic effects targeting oligodendroglial pathology, particularly when integrated into multimodal biomarker frameworks. Importantly, p25α (also known as tubulin polymerization–promoting protein), an oligodendrocyte-specific protein that is normally enriched in myelin but redistributes to the oligodendroglial soma and glial cytoplasmic inclusions in MSA, represents a pathologically proximal marker of disease biology. In post-mortem MSA brain tissues, p25α relocalization from myelin to the cytoplasm precedes and colocalizes with α-synuclein aggregation, consistent with its role in promoting pathological α-synuclein assembly and oligodendroglial degeneration [101, 102]. Experimental evidence further suggests that p25α can influence α-synuclein strain formation and enhance neurodegenerative properties, highlighting its potential as a mechanistically informative biomarker of oligodendroglial pathology [101, 103]. Incorporating p25α-related measures into biomarker panels may therefore enhance sensitivity to disease-modifying effects directed at early oligodendroglial pathology.

**Table 6:** Clinical, imaging, and biomarker outcome measures assessed in completed clinical trials for multiple system atrophy

Drug/NCT No.	Clinical markers	Imaging markers	Biofluid biomarkers	Target-engagement assessment	References
Sirolimus/NCT03589976	UMSARS	MRI: diffusion metrics (MD, FA), advanced microstructural parameters (kurtosis, axonal water fraction, global/regional atrophy fraction) in subset *n* = 15 (sirolimus 11, placebo 4) OCT: RNFL & macular GCC thickness in subset 10 (sirolimus 8, placebo 2)	Plasma NfL, αSyn‑containing CNS‑originated exosomes in subset 14 (11 sirolimus, 3 placebo)	Not assessed	[14]
Rifampicin/NCT01287221	UMSARS, COMPASS-select	Not reported	Not reported	Not assessed	[17]
Lithium/NCT00997672	UMSARS, EQ-5D, BDI-II	Not reported	Not reported	Not assessed	[24]
EGCG/NCT02008721	UMSARS II, CGI-Severity, CGI-Change	MRI: global/regional atrophy, iron deposition in subset 32 (EGCG 17, placebo 15)	Not reported	Not assessed	[30]
Minocycline/NCT00146809	UMSARS II, UPDRS, EQ-5D, SF-12	PET: ^11^C-PK11195 PET (microglial activation) in subset* n* = 8 (minocycline 3, placebo 5)	Not reported	Not assessed	[46]
IVIg/NCT00750867	UMSARS	MRI: global/regional atrophy	Not reported	Not assessed	[48]
Ubiquinol/UMIN000031771	UMSARS I, II, Barthel index, SARA, 10-m walk time	Oxygen PET (No participants)	Not reported	Plasma total CoQ10 levels, mean trough CoQ10 level	[64]
Riluzole/NCT00211224	Survival, H&Y staging, SEADL, CGI-severity, CGI-dysautonomia, SMDS, MMSE	Not reported	Not reported	Not assessed	[68]
Rasagiline/NCT00977665	UMSARS, CGI-I, COMPASS-Select, MSA-QoL, MoCA, BDI, change in anti-parkinsonian/anti-orthostatic hypotension drugs, falls count	MRI: diffusion metrics (MD, FA) in putamen, caudate, globus pallidus, and MCP; structural scores including putaminal abnormalities, putaminal atrophy/hyperintense rim/hypointensity; pontine, cerebellar and MCP atrophy; hot cross bun sign	Not reported	Not assessed	[76]
Fluoxetine/NCT01146548	UMSARS, SCOPA-AUT, BDI, MSA-QoL, mortality	Not reported	Not reported	Not assessed	[83]
Growth Hormone/N/A	UPDRS, UMSARS, HRV during forced respiration; blood pressure response to head‑up tilt	Not reported	Not reported	Not assessed	[91]
Inosine/NCT03403309	UMSARS, MMSE, MoCA, GDS	Not reported	Not reported	Serum uric acid levels	[73]
Exendin-4 (Exenatide)/NCT04431713	UMSARS, COMPASS-Select, UDRS, MSA-QoL, CGI, MoCA, BDI-II, falls diaries, sensor‑derived gait measures	MRI: VBM in subset *n* = 14 (7 exenatide, 7 placebo)	Plasma/CSF NfL, CSF αSyn oligomer load	Serum exenatide levels, CSF exenatide levels	[92]

UMSARS, Unified Multiple System Atrophy Rating Scale; COMPASS-Select, Composite Autonomic Symptom Scale – Select; CGI, Clinical Global Impression; CGI-I, Clinical Global Impression–Improvement; EQ-5D, EuroQol 5-Dimension scale; BDI, Beck Depression Inventory; GDS, Geriatric Depression Scale; MSA-QoL, Multiple System Atrophy Quality of Life scale; MoCA, Montreal Cognitive Assessment; MMSE, Mini-Mental State Examination; SEADL, Schwab and England Activities of Daily Living; SMDS, Strasbourg Multiple System Atrophy Disability Scale; SARA, Scale for the Assessment and Rating of Ataxia; SCOPA-AUT, Scales for Outcomes in Parkinson’s Disease–Autonomic; UPDRS, Unified Parkinson’s Disease Rating Scale; UDRS, Unified Dystonia Rating Scale; H&Y, Hoehn and Yahr stage; HRV, Heart Rate Variability; VBM, Voxel-Based Morphometry; MD, Mean Diffusivity; FA, Fractional Anisotropy; MCP, Middle Cerebellar Peduncle; OCT, Optical Coherence Tomography; RNFL, Retinal Nerve Fiber Layer; GCC, Ganglion Cell Complex; NfL, Neurofilament Light chain; αSyn, alpha-synuclein; CoQ10, Coenzyme Q10; CSF, Cerebrospinal Fluid; N/A, not available

Another key consideration in evaluating therapeutic candidates for MSA is the pharmacokinetic feasibility of delivering the drug to the CNS. In neurodegenerative diseases such as MSA, achieving sufficient concentrations in the brain and CSF is essential for meaningful clinical effect. For instance, sirolimus showed promising efficacy in preclinical MSA models by enhancing autophagy and reducing α-synuclein burden. However, subsequent pharmacokinetic studies have demonstrated that sirolimus exhibits extremely limited CNS penetration, with brain concentrations reported to be less than 1% of plasma levels [104]. Notably, this limitation is further supported by a post-hoc target engagement analysis of neuronal-derived extracellular vesicles in sirolimus-treated patients. No differences in mTOR pathway markers were observed between sirolimus- and placebo-treated groups, suggesting that orally administered sirolimus at the tested doses did not achieve meaningful neuronal target engagement in the CNS [14]. A similar limitation has been observed with nilotinib, another autophagy-modulating agent advanced into clinical testing. In a Phase II PD trial, nilotinib failed to demonstrate clinical benefit and achieved only minimal CSF exposure, with brain-to-plasma concentration ratios of approximately 0.38% at 150 mg (0.94 nM CSF vs. 245.2 nM plasma) and 0.53% at 300 mg (1.6 nM CSF vs. 299.5 nM plasma) [37]. This discrepancy between peripheral and central exposure likely contributed to its failure in clinical trials. At the same time, the strength and specificity of the biological rationale must also be considered—particularly in a disease like MSA, where the precise pathogenic mechanisms remain incompletely defined. Many compounds have been repurposed based on studies in PD models, which primarily reflect neuronal α-synuclein pathology. However, MSA is regarded as a primary oligodendrogliopathy, and therapeutic strategies derived from PD may not sufficiently engage this glial pathology. Importantly, accumulating evidence indicates that the α-synuclein strains present in MSA differ structurally and biologically from those found in PD, exhibiting distinct seeding kinetics, cell-type tropism, and resistance to degradation [105–108]. These strain-specific properties further limit the translational relevance of PD-derived therapeutic approaches and underscore the need for MSA-tailored target engagement.

In this regard, sirolimus and rifampicin are notable in that both underwent preclinical validation in MSA-specific models prior to clinical testing, whereas other agents (e.g., EGCG, nilotinib) were advanced with limited evidence of efficacy in MSA-relevant systems. The lack of robust target engagement within oligodendrocytes—either due to insufficient brain penetration or inadequate model selection—may therefore be an underrecognized contributor to clinical trial failures. For example, in the case of EGCG, in vitro studies have shown that this compound can redirect α-synuclein into off-pathway, non-fibrillar assemblies associated with reduced cytotoxicity in non-neuronal cell assays [109]. However, whether these EGCG-induced intermediates are similarly non-toxic or biologically relevant in neuronal cells or oligodendrocytes has not been demonstrated. Given that oligodendroglial dysfunction is central to MSA pathogenesis [1], reliance on non-glial models or aggregation assays alone may provide an incomplete or misleading assessment of therapeutic potential. Although modelling of MSA poses substantial technical challenges, these considerations underscore the critical importance of validating candidate compounds in preclinical MSA-relevant cellular and animal models as a prerequisite proof-of-concept before advancing to clinical trials in MSA patients. Future development should prioritize compounds with proven activity in MSA-relevant models and ensure adequate CNS bioavailability, ideally supported by early-phase biomarker studies confirming central drug exposure.

Lastly, genetic background that may influence treatment responsiveness is also an important consideration. Variants in *COQ2*, the gene encoding para-hydroxybenzoate-polyprenyl transferase in the CoQ10 biosynthetic pathway, have been identified in both familial and sporadic MSA, particularly among East Asian populations [54]. These variants are associated with reduced CoQ10 synthesis and heightened vulnerability to mitochondrial oxidative stress, providing a mechanistic rationale for the therapeutic efficacy observed with ubiquinol supplementation. Accordingly, systematic genotyping of *COQ2* and related mitochondrial or oxidative-stress pathway genes could enable pharmacogenomic enrichment in future trials, helping identify subgroups most likely to benefit from CoQ10-based or other mitochondrial-targeted therapies. Beyond *COQ2*, incorporating genome-wide or targeted sequencing in MSA cohorts may uncover modifier variants that affect disease progression, treatment response, or subtype predominance (MSA-P vs. MSA-C). Although few studies have examined sex-related differences in MSA, some reports suggest potential variations in clinical presentation [110, 111], which may, however, be masked by the aggressive and rapidly progressive nature of the disease. In the future, it will be important not only to clarify potential sex effects intrinsic to MSA but also to consider possible sex-specific responses to therapy, given that several studies in PD have reported gender-dependent effects of uric acid [112–115]. Although our IMPROVE-MSA trial did not reveal significant differences in outcomes between sexes in stratified analyses [73], further studies are warranted to delineate the role of gender in MSA pathophysiology and treatment response and to incorporate these factors into the design of future precision clinical trials. Integrating genotyping into biomarker-guided trial frameworks will further enhance precision in patient selection, enable ancestry- and sex-informed analyses, and promote harmonization across international MSA studies.

Despite these challenges, several encouraging signals have been reported. A 48-week randomized trial of high-dose ubiquinol in early MSA patients demonstrated significantly smaller declines in UMSARS Part II scores compared to placebo, marking the first instance of a repurposed compound achieving its primary endpoint in MSA [64]. An open-label randomized trial of exenatide also reported a smaller increase in UMSARS scores compared with standard care, although the absence of corresponding biomarker changes and the non-blinded design limit conclusions regarding disease-modifying effects [92]. Inosine, though tested only over 24 weeks, successfully elevated serum urate levels and demonstrated feasibility for future longer studies [73]. Another example of a potentially immune-modulating approach is IVIg. In a small open-label pilot study, IVIg also showed preliminary improvements in UMSARS scores, though methodological limitations precluded definitive conclusions [48]. Nonetheless, the biological rationale for immune-targeted or anti-inflammatory strategies remains compelling, given the accumulating evidence of glial and microglial activation in MSA pathology. Taken together, these findings underscore that meaningful therapeutic effects may be achievable with optimized trial designs, earlier patient recruitment, and longer follow-up durations.

Another important lesson is the recognition that MSA pathogenesis is multifactorial. Past repurposing attempts often targeted a single pathway—such as α-synuclein aggregation, neuroinflammation, or oxidative stress in isolation. However, MSA involves all of these processes in parallel. The limited efficacy of single-agent approaches, many borrowed from PD, suggests that monotherapies may be insufficient to meaningfully alter disease progression once the pathogenic cascade is underway. Future development efforts should explore rationally designed combination regimens and trial designs that capture this complexity, including strategies that target both neuronal and glial pathology simultaneously.

## Conclusion and future directions

Looking forward, the field of MSA therapeutics is entering a new stage that integrates lessons learned from past failures. Future trials must prioritize earlier intervention, ideally enrolling patients at prodromal or possible MSA stages, and adopt longer follow-up durations to detect disease-modifying effects. Biomarker-guided trial designs are essential; neurofilament light chain, α-synuclein seeding assays, and advanced MRI markers may help identify patients at a higher risk of rapid progression, thereby enriching cohorts and increasing statistical power. Improved training of investigators in standardized clinical scales, along with adaptive designs and interim futility analyses, can further enhance trial efficiency. In parallel, establishing a multicenter, longitudinal MSA cohort—analogous to the PPMI initiative in PD—should be a key collective goal. Such a network would enable the definition and validation of standardized progression trajectories under real-world, harmonized conditions. These trajectories could then serve as benchmark references for future interventional studies, allowing efficacy signals to be interpreted relative to the natural history of the disease. This strategy, recently exemplified by the PASADENA study in PD [116], could provide a practical framework to evaluate disease-modifying effects in MSA despite its rarity and rapid progression. Nevertheless, the absence of placebo effects in natural history cohorts represents an important limitation, as efficacy derived from comparisons with single-arm trials may be systematically overestimated. Accordingly, the use of such cohorts should be complemented by objective, placebo-insensitive biomarkers including fluid and imaging measures rather than solely clinical scales.

In terms of pharmacological strategies, novel approaches are under active investigation. Immunotherapies targeting α-synuclein, antisense oligonucleotides, and iron-chelating agents such as ATH434 represent promising avenues [117]. At the same time, repurposed compounds with encouraging signals—such as ubiquinol, inosine, and IVIg—warrant validation in larger, biomarker-enriched, placebo-controlled trials. Importantly, given the multifactorial nature of MSA pathogenesis, future strategies should not be restricted to monotherapy. Combination regimens that simultaneously target oxidative stress, neuroinflammation, and α-synuclein aggregation may offer synergistic neuroprotection. Based on current evidence, several rational combinations can be envisioned. For instance, pairing a mitochondrial support agent such as ubiquinol with an anti-inflammatory or immune-modulating therapy (e.g., sirolimus or IVIg) could concurrently mitigate oxidative stress and glial activation. Similarly, combining a protein-aggregation inhibitor (such as EGCG or future immunotherapeutic approaches) with a remyelination-enhancing compound (e.g., benztropine or sobetirome) might address both proteostatic and glial dysfunction. Clinical testing of such combinations, perhaps through platform or factorial trial designs, represents a logical next step.

In summary, while no repurposed drug has yet achieved regulatory approval for MSA, the cumulative evidence provides valuable guidance for the next generation of trials. By addressing trial design barriers, incorporating biomarker-based patient selection, and exploring rational combination therapies, the field may finally move closer to achieving disease modification in this devastating disorder.

## Data Availability

No datasets were generated or analyzed during the current study. All information summarized here is derived from previously published sources cited in the manuscript.

## Abbreviations

ADL: Activities of daily living
AE: Adverse event
ALS: Amyotrophic lateral sclerosis
BDI: Beck depression inventory
BDNF: Brain-derived neurotrophic factor
CGI: Clinical Global Impression
CI: Confidence interval
CNS: Central nervous system
CoQ10: Coenzyme Q10
COMPASS: Composite Autonomic Symptom Score
CSF: Cerebrospinal fluid
EQ-5D: EuroQol 5-dimension questionnaire
GCI: Glial cytoplasmic inclusion
GDNF: Glial cell line–derived neurotrophic factor
GH: Growth hormone
HR: Hazard ratio
HRV: Heart rate variability
IGF-1: Insulin-like growth factor-1
IVIg: Intravenous immunoglobulin
MAO-B: Monoamine OXIDASE-B
MMSE: Mini-Mental State Examination
MoCA: Montreal Cognitive Assessment
mTOR: Mammalian target of rapamycin
MSA: Multiple system atrophy
MSA-C: Cerebellar subtype of MSA
MSA-P: Parkinsonian subtype of MSA
NfL: Neurofilament light chain
NMDA: N-methyl-D-aspartate
PET: Positron emission tomography
QoL: Quality of life
RCT: Randomized Controlled Trial
r-hGH: Recombinant human growth hormone
SCOPA-AUT: Scales for Outcomes in Parkinson’s Disease–Autonomic
SSRIs: Selective Serotonin Reuptake Inhibitors
UMSARS: Unified Multiple System Atrophy Rating Scale
UPDRS: Unified Parkinson’s Disease Rating Scale
